# The p.Ile202Thr Substitution in TUBB2B Can Be Associated with Syndromic Presentation of Congenital Fibrosis of the Extraocular Muscles

**DOI:** 10.3390/genes16101182

**Published:** 2025-10-11

**Authors:** Cecilia Mancini, Luigi Chiriatti, Alessandro Bruselles, Paola D’ambrosio, Andrea Ciolfi, Marco Ferilli, Camilla Cappelletti, Mattia Carvetta, Francesca Clementina Radio, Viviana Cordeddu, Marcello Niceta, Marta Parrino, Rossella Capolino, Corrado Mammì, Rossana Senese, Mario Muto, Manuela Priolo, Marco Tartaglia

**Affiliations:** 1 Molecular Genetics and Functional Genomics, Bambino Gesù Children’s Hospital, IRCCS, 00143 Rome, Italy; cecilia.mancini@opbg.net (C.M.); francesca.clementina.radio@gmail.com (F.C.R.);; 2 Department of Oncology and Molecular Medicine, Istituto Superiore di Sanità, 00161 Rome, Italyviviana.cordeddu@iss.it (V.C.); 3 Medical and Molecular Genetics, AORN A. Cardarelli, 80131 Naples, Italy; 4 Department of Computer, Control and Management Engineering, Sapienza University, 00185 Rome, Italy; 5 Department of Biomedicine and Prevention, University “Tor Vergata”, 00173 Rome, Italy; 6 Department of Biochemical Sciences “Alessandro Rossi Fanelli”, Sapienza University, 00185 Rome, Italy; 7 Rare Diseases and Medical Genetics Unit, Bambino Gesù Children’s Hospital, IRCCS, 00165 Rome, Italy; 8 Medical Genetics Unit, GOM Bianchi-Melacrino-Morelli, 89124 Reggio Calabria, Italy; 9 Department of Advanced Diagnostic and Therapeutic Technologies, AORN A. Cardarelli Hospital, 80131 Naples, Italy

**Keywords:** *TUBB2B*, tubulinopathies, CFEOM, CDCBM7, malformations of cortical development, WGS, DNA methylation profiling

## Abstract

**Background**: Dominantly acting variants in *TUBB2B* have primarily been associated with cortical dysplasia complex with other brain malformations 7 (CDCBM7), a disorder in which cortical brain abnormalities are typically linked to developmental delay/intellectual disability (DD/ID) and seizures. While the majority of *TUBB2B* pathogenic variants have been linked to isolated CDCBM7, only one family with CDCBM7 and congenital fibrosis of the extraocular muscles (CFEOM) has been reported so far. We describe a second individual with a severe phenotype of CFEOM combined with CDCBM7 carrying a pathogenic *TUBB2B* missense variant previously reported in two individuals with isolated CDCBM7. **Methods**: A trio-based WGS analysis was performed. The structural impact of the identified substitution was assessed by using the UCSF Chimera (v.1.17.3) software and PyMOL docking plugin DockingPie tool. **Results**: WGS analysis identified a de novo missense *TUBB2B* variant (p.Ile202Thr, NM_178012.5), previously associated with isolated CDCBM7. Structural analysis and docking simulations revealed that Ile202 contributes to establishing a proper hydrophobic environment required to stabilize GTP/GDP in the β-tubulin pocket. p.Ile202Thr was predicted to disrupt these interactions. **Conclusions**: Our findings broaden the mutational spectrum of *TUBB2B*-related CFEOM, targeting a different functional domain of the protein, and further document the occurrence of phenotypic heterogeneity. We also highlight the limitations of exome sequencing in accurately mapping *TUBB2B* coding exons due to its high sequence homology with *TUBB2A* and suggest targeted or genome analyses when clinical suspicion is strong.

## 1. Introduction

Tubulinopathies are a wide and clinically overlapping group of neurodevelopmental disorders (NDDs) mainly characterized by malformations of cortical development (MCDs). These conditions are frequently associated with brain size abnormalities, cognitive and motor impairment, abnormal muscular tone, epilepsy, and craniofacial features (i.e., retrognathism, hypertelorism, and progressive facial diplegia) [1,2]. MCDs have recently been reclassified into three major “patterns”: (a) the lissencephaly pattern, characterized by significant thickening of the cortex; (b) the micro-lissencephaly pattern, referring to the most severe form of cortical dysgenesis combining extreme congenital microcephaly and lissencephaly; and (c) the dysgyric pattern, referring to a cortex of normal thickness but with an abnormal gyral pattern characterized by malformations of sulcal depth or orientation [1,2,3]. α-tubulin and β-tubulin are 55-kD polypeptides that polymerize as heterodimers to form microtubules, a major component of the cytoskeleton [4]. The microtubules assembly process is tightly regulated by GTP binding to the β-tubulin subunit. The GTP binding site on the β-tubulin surface is located in a pocket, which is primarily formed by highly conserved hydrophobic and aromatic residues, establishing the optimal environment for the proper fitting of the non-polar surface of the GTP guanine ring and its stabilization through a network of hydrophobic interactions [4,5]. The GTP-bound state stabilizes the α-β tubulin heterodimer, allowing its incorporation into the dynamic plus-end part of the growing microtubules [6]. The addition of GTP-bound α/β tubulin dimers is energetically favorable and occurs rapidly, enabling cells to build complex microtubule networks. On the other hand, GTP hydrolysis into GDP causes a conformational shift of the heterodimer, leading to its disassembly from the microtubule minus-end. The GTP-to-GDP transition is a critical process for the regulation of microtubule dynamics, coordinating the switch between growth and shrinkage phases as well as cellular adaptability and plasticity [4,5]. As a proof, defects in GTP/β-tubulin binding impair the finely tuned regulation of microtubule dynamics, affecting cell shape maintenance, mitotic spindle assembly, and control of intracellular transport and cell migration [5,7,8,9].

In the developing brain, tubulins are critical for neuronal processes including proliferation, migration, differentiation, cortical organization, and axonal guidance [7,10,11,12]. Pathogenic heterozygous missense variants in genes encoding brain-expressed tubulins (i.e., *TUBA1A, TUBA3E, TUBA4A, TUBA8, TUBB2A, TUBB2B, TUBB3, TUBB4A,* and *TUBG1*) have been identified as the molecular causes underlying various MCDs [1,2,13]. Among these, dominant *TUBB2B* variants have been associated with cortical dysplasia, complex, with other brain malformations 7 (CDCBM7, MIM #610031), a condition characterized by a broad range of MCDs with developmental delay/intellectual disability (DD/ID) and seizures [1,14]. The majority of reported individuals affected with CDCBM7 carry missense variants as de novo events. A few families with multiple affected members have also been described, who are generally characterized by a milder clinical presentation and wide intrafamilial variability [1,2,14]. Disease-causing variants have been demonstrated to impair neuronal migration, leading to a wide spectrum of MCDs, whose severity directly correlates with the degree of motor and cognitive deficits [1,3].

*TUBB2B* and *TUBB3*-related β-tubulinopathies can also present as disorders of axon growth and guidance, causing additional anomalies in axons innervating the oculomotor muscles, resulting in an eye-movement disorder termed as congenital fibrosis of the extraocular muscles (CFEOM; MIM: PS135700) [2]. CFEOM is mainly caused by dominantly acting variants in *KIF21A* and *TUBB3* genes [2,13,15,16,17,18,19,20]. While variants in *KIF21A* usually cause isolated CFEOM (CFEOM1; MIM: 135700) [15,21], those in *TUBB3* are associated with either isolated or syndromic CFEOM (CEOM3A MIM: 600638), in which additional features (e.g., DD/ID, learning disabilities, and brain structural defects) occur, and complex cortical dysplasia with other brain malformations (CDCBM1; MIM: 614039) [16,22]. To date, a few unrelated affected individuals with heterozygous pathogenic variants in *TUBA1A* have been reported [20], while a single family with CDCBM7 and CFEOM has been described to carry a pathogenic missense variant (p.Glu421Lys) in *TUBB2B* [17]. Clinically, CFEOM results in a non-progressive ophthalmoplegia with or without ptosis affecting the oculomotor nerve [22]. Affected subjects show severe limitation of vertical gaze, usually affecting upgaze, and variable impairment of horizontal gaze. Typically, individuals with CFEOM compensate for the ophthalmoplegia by holding improper head positions at rest and tracking objects with their heads rather than their eyes [23]. Binocular vision is typically absent, and refractive errors are frequently observed; the Marcus-Gunn phenomenon, characterized by rhythmic upward jerking of their upper eyelid during suction, also occurs [23].

Here, we describe a second unrelated individual presenting with a severe phenotype of CFEOM combined with CDCBM7, carrying a missense (p.Ile202Thr) pathogenic *TUBB2B* substitution, which had previously been associated with isolated CDCBM7 [13]. Our findings expand the mutational spectrum of the *TUBB2B*-related presentation of CDCBM7 with CFEOM, involving a different functional domain of the protein, and further document the occurrence of phenotypic heterogeneity. We also highlight the poor ability of exome sequencing (ES) in properly mapping reads covering *TUBB2B* coding exons due to its high sequence homology with *TUBB2A* and recommend the use of alternative targeted (i.e., Sanger sequencing) or genomic (whole genome sequencing (WGS)) analyses in the presence of a strong clinical suspicion.

## 2. Materials and Methods

The subject was enrolled in a research program directed to understand the molecular causes of unclassified pediatric disorders at the Ospedale Pediatrico Bambino Gesù, Rome, Italy. The study was approved by the local Institutional Ethical Committee (protocol code 409 RF-2021-12374963, 5 April 2023). Clinical data, pictures, neuroimaging, and blood samples were collected, used, and stored after signed informed consents from his parents were secured, in accordance with the ethical standards outlined in the Declaration of Helsinki. Permission to publish the clinical pictures was obtained.

### 2.1. Exome Sequencing Data Analysis

ES was performed on leukocyte-derived DNA samples using a trio-based approach. Sequencing was run on NovaSeq6000 platform (Illumina, San Diego, CA, USA), and the SureSelect QXT Human All Exon V7 kit (Agilent, Santa Clara, CA, USA) was used for target region enrichment. The raw data were processed applying an internal pipeline as previously delineated [24,25,26] according to recently reported best practices [27], applying a periodic re-evaluation step based on GenomeAlert! and Amelie frameworks [28,29]. The UCSC GRCh37/hg19 genome assembly was used as reference for read alignment using the BWA-MEM v0.7.12 tool [30], and variant calling was performed with HaplotypeCaller (GATK v3.7) [31]. SnpEff v.5.0 and dbNSFP v.4.2 tools were used for variants’ functional annotation [32,33]. Variants with MAF greater than 0.001 were excluded from both public (dbSNP150 and gnomAD v.4.1.0) and in-house databases (approximately 3350 exomes). Mendelian Clinically Applicable Pathogenicity (M-CAP) v.1.3 [34], Combined Annotation Dependent Depletion (CADD) v.1.6 [35], and InterVar v.2.0.1 [36] were considered for functional impact prediction.

### 2.2. DNA Methylation Profiling Analysis

Genomic DNA was extracted from peripheral blood using standard techniques. Bisulfite conversion was performed, and samples were processed using the Infinium Methylation EPIC BeadChip v.1/v.2 (Illumina), according to the manufacturer’s protocol. DNA methylation (DNAm) was analyzed using a previously established pipeline [37,38]. Data were grouped based on the currently available DNAm signature for Kabuki syndrome [39,40] through hierarchical clustering and multidimensional scaling, applying pairwise Euclidean distances between samples. The training model of the Support Vector Machine classifier was carried out as previously reported [41].

### 2.3. RNA Analysis

The proband’s peripheral blood was collected using PAXgene Blood RNA Tubes (Qiagen, Mannheim, Germany). Total RNA was extracted using PAXgene Blood RNA Kits (Qiagen), and cDNA was obtained using SuperScript IV Reverse Transcriptase (Thermo Fisher Scientific, Waltham, MA, USA), following manufacturer’s instructions. A specific PCR-based assay and direct sequencing were performed to investigate the impact of the c.564+4G>T variant on the *KDM6A* transcript process.

### 2.4. WGS Data Analysis

Trio-based WGS data were obtained using a 2 × 150 bp paired-end read protocol on a NovaSeq 6000 platform (Illumina, San Diego, CA, USA) obtaining 30x median coverage. Base calling and data analysis were carried out using Bcl2FASTQ (Illumina). Paired-end reads were mapped to the GRCh38 reference sequence; small variant calling and joint genotyping were performed using Sentieon v.2023-08 (https://www.sentieon.com, accesed on 21 January 2025). SNPs and indels hard filtering were applied using HaplotypeCaller (v. 3.8.0) [31]. High-quality variants were filtered by frequency ≤ 5% using an in-house WGS population-matched database (>350 WGS). Remaining coding sequence variants were annotated and filtered using a custom pipeline, while variants in non-coding regions were annotated and prioritized using Genomiser (data v.2309) [42]. Structural variants were identified using DELLY v.1.1.6 [43] and prioritized using AnnotSV v.3.4. [44]. The identified *TUBB2B* variant was validated by bidirectional Sanger sequencing.

### 2.5. Structural and Docking Analysis

The wild-type TUBB2B structure (PDB ID: 6E7C) [45], complexed with a non-hydrolyzable GTP analog (phosphomethylphosphonic acid guanylate ester, GMPcPP), was used to assess the structural consequences of the isoleucine-to-threonine substitution at codon 202 in the hydrophobic GTP binding pocket, using the UCSF Chimera software v.1.17.3 (https://www.cgl.ucsf.edu/chimera/, accessed on 11 March 2025) [34]. DockingPie v.1.0.1 [46], a docking plugin for PyMOL v.2.7 (Schrödinger LLC, New York, NY, USA), in combination with the AutoDock Vina v.1.2.3 scoring function [47,48], was used to perform a total of 200 docking poses. We calculated pose ranking and affinity using the GMPcPP as ligand and wild-type (N = 100) or p.Ile202Thr (N = 100) TUBB2B as rigid structure.

## 3. Results

### 3.1. Clinical Findings

The proband is a 3-year-old male, second child of non-consanguineous parents. The maternal lineage was notable for epilepsy in the grandfather and the alpha-thalassemia trait in multiple members. The pregnancy was spontaneously conceived but complicated by placental abruption during the first trimester with mild intrauterine growth restriction. Labor was induced at 41 weeks and 4 days. Auxological parameters at birth were as follows: weight 3.18 kg (−1.24 SD), length 50 cm (0.93 SD), and head circumference 33 cm (−1.95 SD). Apgar scores were 9 and 10 at one and five minutes, respectively. He suffered from perinatal fracture of the right clavicle. At birth, bilateral ptosis and axial hypotonia were noticed.

He came to our observation at 1 year because of delay in psychomotor development, hypotonia, and dysmorphic facial features. Growth parameters were as follows: weight 11 kg (0.54 SD), height 79 cm (1.12 SD), and OFC 43 cm (−2.7 SD). He presented with microcephaly, brachycephaly with plagiocephaly, and a prominent metopic ridge. Major facial features were long palpebral fissures, bilateral epicanthus and severe palpebral ptosis, thick eyebrows, broad nasal bridge, low-set and posteriorly rotated ears, anteverted nares, long and smooth philtrum, drooling and high-arched palate. Dorso-lumbar scoliosis with left convexity and dorsal kyphosis, and left cryptorchidism were also reported. He achieved head control at 1.5 years, began rolling at 2 years, and started standing with the aid of braces at 2 years and 6 months. At 3 years, communication has been limited to vocalizations as per 1 year of age, and needs were expressed through gestures. Dentition was delayed, and sphincter continence was not achieved at 3 years. He underwent corrective surgery for right ptosis at 1 year and 10 months with scarce benefit. At 2.5 years, an atrophic left testicular remnant was removed via orchidopexy. Cardiac assessment identified an atrial septal defect.

**Figure 1:**
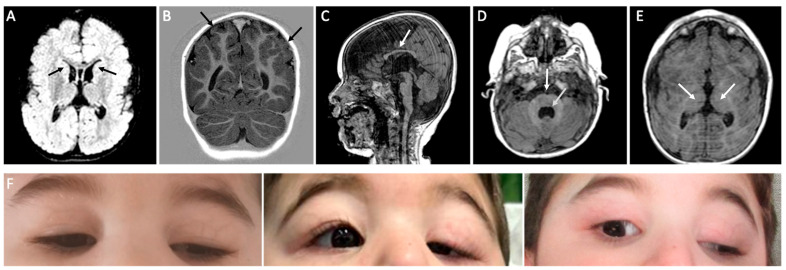
Brain MRI and clinical pictures of the proband. (**A**) Flair sequence, protrusion of the head of the caudate nuclei into the lateral ventricles giving the frontal horns a hooked configuration. (**B**) T1-w. Polymicrogyria-like cortical dysplasia predominantly at the fronto-parietal convexities bilaterally. (**C**) T1-w. Thin corpus callosum. (**D**) T1-w. Hypoplasia of the pons with central fissure and dilatation of the IV ventricle, dysmorphic appearance of cerebellar peduncles. (**E**) T1-w. Globular appearance of thalami. (**F**) Severe bilateral ptosis resulting from CFEOM is shown at different ages: 8 months (left, presurgical), 1.5 years (center, postsurgical, right eye), and 3.5 years (right, postsurgical right eye).

Brain MRI scan at 1 year revealed cortical dysplasia, polymicrogyria (predominantly at the fronto-parietal convexities, bilaterally), and dysgenesis of the anterior limb of the internal capsule, bilaterally, resulting in a round, fused appearance of the caudate and putamen. The caudate’s heads protruded into the lateral ventricles, resulting in hooked frontal horns with a globular thalamus. Slight dilation of the supratentorial ventricular system, particularly posteriorly, was noticed together with residual septum pellucid cyst, *cavum vergae*, thin corpus callosum, hypoplasia of the pons with central fissure, and IV ventricle dilation (Figure 1). The middle cerebellar peduncles had a dysmorphic appearance, with hypoplasia of the cerebellar vermis and globular appearance of the bulb. Hypoplasia of extraocular muscles was noted, bilaterally. A second MRI performed at 2 years and 6 months (images not available) confirmed occurrence of hypoplasia of the corpus callosum.

At last examination (3 years), he presented with the following auxological parameters: weight 17.5 kg (1.65 SD), height 98 cm (0.56 SD), and OFC 46.5 cm (−1.87 SD). Clinical evaluation revealed lack of visual engagement and inability to follow simple commands, although the child reacted to visual and auditory stimuli. Axial hypotonia was present, and posture was supported by compensatory mechanisms. The antigravity movements of limbs and trunk were present, and he could sit independently. Facial features included bilateral ptosis, more pronounced on the left, esotropia with limited upward gaze, Marcus-Gunn sign during suction, high-arched palate, sialorrhea, and dental diastema. Joint mobility was preserved, but muscle strength was not evaluable due to hypotonia. Deep tendon reflexes were hyperactive in the limbs.

### 3.2. Molecular Findings and Structural Analysis

Due to an uninformative clinical exome performed in the routine diagnostic setting, a trio-based ES analysis was conducted in the context of a research program focused on molecular unsolved pediatric disorders. A single private (gnomAD v4.1.0) hemizygous intronic variant (c.564+4G>T) of the *KDM6A* gene (NM_001291415.2) was identified. *KDM6A* pathogenic variants have been associated with the Kabuki syndrome (KS, MIM #300867), a congenital disorder characterized by DD/ID, typical facial features (i.e., ptosis and strabismus), minor skeletal anomalies, congenital heart defects, genitourinary anomalies, postnatal growth deficiency, and, rarely, polymicrogyria [49]. The variant was inherited from the carrier mother and classified as a variant of uncertain significance (VUS) according to ACMG/AMP criteria [50]. To evaluate the effect of the variant on transcript integrity, we first performed an in silico study with SpliceAI [51], which resulted negative. To rule out the clinical relevance of the c.564+4G>T variant, we tested his genome-wide DNAm profile for the KS-specific episignature [37,40]. The probeset (covering 153 genomic sites) was used to classify the DNAm profile and compared two in-house reference cohorts, composed by 14 individuals with molecularly confirmed KS (“*KMT2D* training set”) and 440 controls (290 healthy subjects and 150 individuals affected with different rare disorders). Unsupervised and supervised analyses consistently grouped the tested individual with controls (Supplemental Figure S1), thereby excluding the clinical relevance of the *KDM6A* variant on the proband’s phenotype in the context of KS. This finding was also confirmed by cDNA analysis that excluded any effect on transcript processing (Supplemental Figure S2).

**Figure 2:**
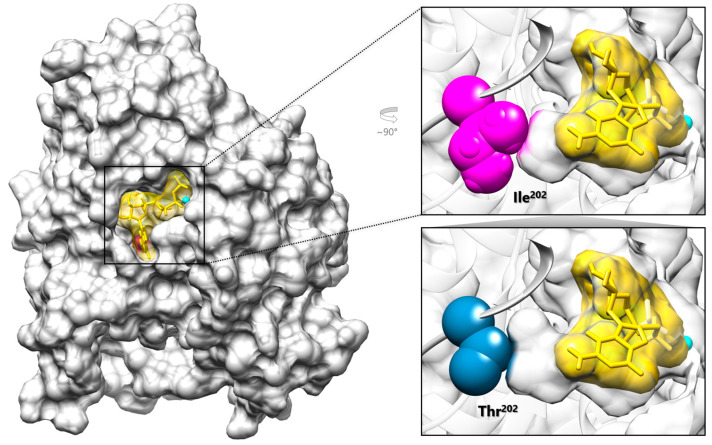
Graphical representation of TUBB2B complexed with the GTP analog GMPcPP. The structural model of TUBB2B (PDB ID: 6E7C) (gray) is shown complexed with the Mg^2+^ ion (cyan) and GMPcPP (yellow, stick and surface representation), which localize within the GTP binding pocket. In the top right panel, Ile^202^ (magenta) is located at the innermost end of the GTP binding pocket together with GMPcPP (**right, top**); the introduced Thr^202^ (blue) is expected to cause a local rearrangement of the pocket (**right, bottom**).

In absence of other candidate variants, we proceeded with a trio-based WGS analysis which revealed a de novo variant in *TUBB2B* (NM_178012.5:c.605T>C), located in exon 4 and predicting an isoleucine-to-threonine substitution at codon 202 (p.Ile202Thr). The affected residue maps within the GTP/GDP purine ring binding site on the β-tubulin surface at the distal end of the binding pocket (Figure 2). The variant had a CADD phred score of 26.9; AlphaMissense predicted a deleterious impact on protein folding (score 0.97), and Aggregated Prediction confirmed a pathogenic effect (score 0.99). According to the ACMG criteria, the variant was classified as pathogenic (class 5). The p.Ile202Thr missense change had not previously been reported in the gnomAD v4.1.0 population database, although it had been described in two individuals with a clinical presentation of CDCBM7 [13], fitting the third class of MCDs [2], as observed in our subject. Retrospectively, the manual inspection of genomic alignments revealed extensive low-quality mapped reads from ES data, affecting *TUBB2A/TUBB2B* homologous regions and causing the filtering out of the *TUBB2B* variant due to low quality issues. The root mean square mapping quality (MQ) over all the reads at the site was 39.41, which is slightly lower than the recommended filtering cut-off of 40 [31]. On the other hand, the more homogeneous coverage of the genomic region from WGS allowed a more robust alignment (MQ = 52.75) and proper variant calling, even using short-read sequencing technology.

Ile^202^ is a branched amino acid with a non-polar hydrophobic side chain that plays a critical role, together with other residues exhibiting similar chemical and structural properties, in establishing the optimal environment for proper GTP/GDP stabilization within the β-tubulin pocket (Figure 2). Although not directly binding to GTP, the isoleucine substitution at codon 202 with threonine, a polar residue, is expected to disrupt the hydrophobic interactions within the pocket, likely affecting GTP stabilization by reducing its binding affinity (Figure 2). To further explore this hypothesis, molecular docking predictions of GMPcPP to either wild-type and p.Ile202Thr TUBB2B were calculated. Compared to the WT protein (mean energy = −7.527 ± 0.615 kcal·mol^−1^, best ranking pose = −9.468 kcal·mol^−1^), the mutant showed a lower binding affinity (mean energy = −6.422 ± 0.645 kcal·mol^−1^, best ranking pose = −8.611 kcal·mol^−1^), with a difference that proved to be statistically significant (*t* (198) = −12.396, *p* < 0.001, |*d*| = 1.753), supporting a local rearrangement of the pocket possibly impacting GTP/GDP binding.

**Figure 3:**
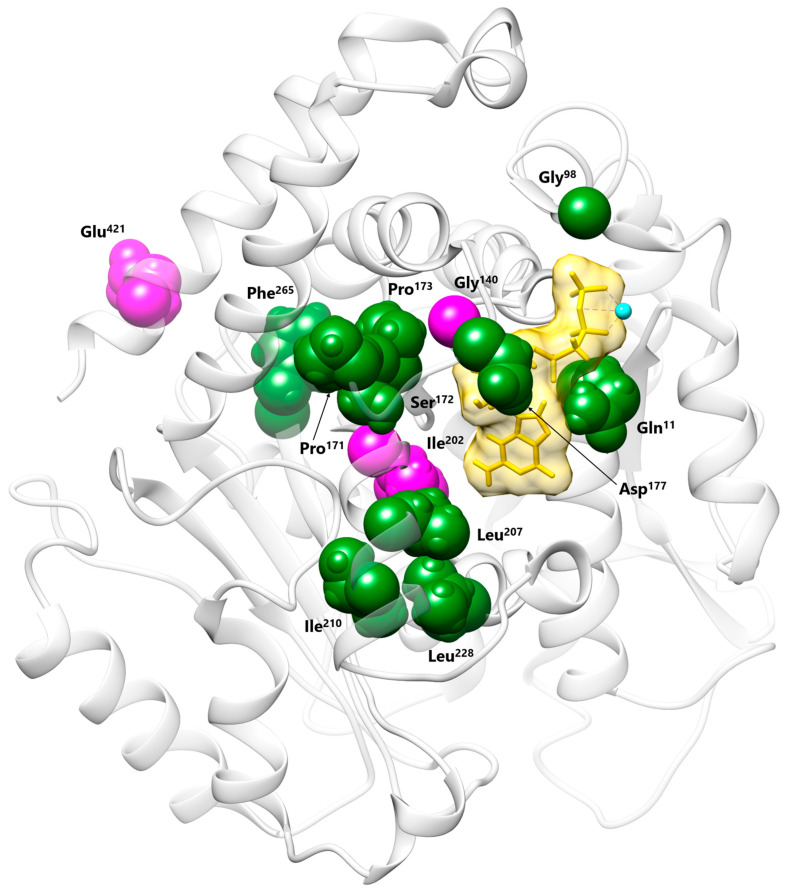
Structural visualization of the TUBB2B residues affected by CDCBM7 and/or syndromic CFEOM disease-causing variants. Most residues map to the GTP/GDP binding pocket. Pathogenic variants associated with isolated CDCBM7 (Gln^11^, Gly^98^, Pro^171^, Ser^172^, Pro^173^, Asp^177^, Leu^207^, Ile^210^, Leu^228^, Phe^265^) are in dark green, while those previously associated with syndromic CFEOM (Gly^140^, Glu^421^) and the presently identified Ile^202^ are in magenta. Conversely, Glu^421^ localizes at the *C*-terminal H12 α-helix and is involved in the kinesin–microtubule interaction.

Among the twelve disease-causing *TUBB2B* missense changes encompassing the GTP binding pocket reported so far, eleven have been associated with isolated CDCBM7 (Figure 3) [1,11,14]. Of these, the p.Gln11Leu and the p.Asp177Val changes are the only two reported substitutions involving residues that directly H-bond the GDP/GTP. The remaining variant, p.Gly140Ala, was described as causative of CDCBM7 with right-sided ptosis, exotropia, and ophthalmoplegia [52], findings consistent with a diagnosis of unilateral CFEOM, although this diagnosis was not explicitly stated in the original report (Figure 3). Of note, the clinical presentation of the present subject showing CDCBM7 with CFEOM had previously been described in a familial case carrying the p.Glu421Lys substitution [17]. The same amino acid substitution had also been identified in an additional individual reported to have CDCBM7 and ptosis [53], a clinical phenotype that likely suggests a diagnosis of CDCBM7 with CFEOM. This residue localizes at the *C*-terminal H12 α-helix, playing a crucial role in kinesin–microtubule interaction (Figure 3) [6]. Based on this evidence, the TUBB2B amino acid substitutions causative of syndromic CFEOM (i.e., p.Ile202Thr, p.Glu421Lys, and likely p.Gly140Ala) hit different domains of the protein and are predicted to have distinct structural/functional impact, though possible functional convergence cannot be ruled out and requires experimental in vitro and/or in vivo analyses. (Figure 3).

## 4. Discussion

Here, we report on an individual with a severe clinical presentation of CDCBM7 with CFEOM carrying the p.Ile202Thr pathogenic substitution of TUBB2B, which had previously been reported to be associated with CDCBM7 [2,13]. Extensive WGS data analysis did not identify any additional clinically relevant variants that could contribute to the observed phenotype, further supporting the causative role of the p.Ile202Thr variant underlying CFEOM. A single family with a clinical presentation of CFEOM and a mild form of CDCBM7 (ID ranging from borderline to moderate) had previously been reported [17,54]. In that family, the *TUBB2B* missense change affected a residue at the *C*-terminus of the protein in three affected members. An extensive literature review revealed two additional subjects carrying disease-causing *TUBB2B* variants and exhibiting CDCBM7, who could retrospectively be considered affected with syndromic CFEOM: the first subject was heterozygous for the p.Gly140Ala substitution and presented with right-sided ptosis, exotropia and ophthalmoplegia; the second subject carried the p.Glu421Lys change and presented with ptosis [52,53]. These findings suggest that mild CFEOM signs might be overlooked in these patients carrying pathogenic *TUBB2B* variants. Radiologically, these individuals, including the present subject, presented with polymicrogyria. Gly^140^, Ile^202^, and Glu^421^ localize in different regions of the β-tubulin structure, and their pathogenic substitutions are predicted to have different functional impacts. Specifically, p.Gly140Ala affects the first residue of a highly conserved glycine-rich region (residues 137 to 146) located upstream of the GTP binding site, which is crucial for ensuring the flexible nature of the loop required to regulate nucleotide access to the binding site. The substitution of Gly^140^ with an alanine was demonstrated to alter the loop conformation, reducing its flexibility and impairing heterodimer formation [52]. On the other hand, Glu^421^ is a critical residue located at the *C*-terminal H12 α-helix, required for proper kinesin–microtubule interactions [6]. Ile^202^ plays a crucial role in establishing the optimal environment for proper GTP stabilization within the β-tubulin pocket. The docking prediction of GMPcPP to the mutated β-tubulin supports the pathogenic impact of the variant, documenting a reduction in the ligand affinity compared to the wild-type protein. Together, our findings expand the mutational spectrum underlying the clinical phenotype of CDCBM7 combined with CFEOM to other TUBB2B functional domains. Experimental studies are required to dissect the consequences of individual amino acid substitutions on TUBB2B function and microtubule dynamics.

Although genotype–phenotype correlations with isolated or syndromic CFEOM have been demonstrated for specific *TUBB3* variants, such associations have remained elusive for tubulinopathies in general, as the collected clinical and molecular information documents that individuals harboring the same pathogenic variant can show diverse clinical manifestations [1,55]. This variability has been observed for the majority of pathogenic variants involving tubulin genes, including *TUBB3*, which is responsible for approximately one-third of CFEOM reported cases [55]. Initially, two separate clusters of *TUBB3* pathogenic variants had been proposed to specifically be associated with CDCBM (e.g., p.Arg46Gly, p.Gly82Arg, p.Thr178Met, p.Glu205Lys, p.Glu288Lys, p.Ala302Val, p.Met323Val, p.Pro357Leu, p.Met388Val) or CFEOM (e.g., p.Arg262Cys, p.Arg262His, p.Arg380Cys, p.Glu410Lys, p.Asp417Asn, and p.Asp417His) [54]. Indeed, the p.Gly71Arg and p.Gly98Ser substitutions were described as the first variants to be associated with a clinical presentation of MCD combined with CFEOM [55]. Recently, an individual carrying the recurrent p.Gly98Ser pathogenic change in *TUBB3* presenting with an isolated form of CDCBM without CFEOM has been reported, revealing that this substitution can underlie both phenotypes [56]. In line with these findings, p.Ile202Thr can be associated with CDCBM7 combined with CFEOM or with isolated CDCBM7. Of note, the spectrum of clinical presentations caused by pathogenic *TUBB2B* variants appears to be broader, ranging from very mild phenotypes to severe ones, including juvenile-onset dystonia as major signs with wide inter- and intra-familial variability reported [14,17,57,58]. Other modifying factors might contribute to the phenotypic expression and likely explain such unpredictability. Exception is confined to a recessive disorder, Uner Tan syndrome, also known as cerebellar ataxia, impaired intellectual development, and disequilibrium syndrome (CAMRQ, MIM #224050), which has been linked to the homozygous pathogenic missense variant (p.Arg390Gln) in *TUBB2B* in one single family [59]. Specifically, this variant has been proven not to affect β-tubulin folding or assembly into the α-β-heterodimer, which is indicative of a mild impact on microtubule function. In this family, carrier parents were unaffected and did not show any ocular sign/feature.

We note that there is evidence that CFEOM (either isolated or syndromic) can be associated with widespread orbital dysinnervation, which can include optic nerve hypoplasia/atrophy (OH/OA) [22,60,61,62,63]. The revision of previously published *TUBB2B* cohorts revealed at least four individuals presenting with OH/OA [11,14,52,63]. This supports the idea that the phenotypic spectrum associated with CFEOM is broader than previously recognized, extending beyond the motor nerves, as originally suggested [62]. Further studies are required to establish whether OH/OA represent additional clinical signs related to CFEOM.

In the present individual, the molecular diagnosis was achieved through WGS, this variant being initially missed due to technical limitations of the targeted approach (clinical exome panel) and ES analyses. *TUBB2B* is characterized by high-sequence homology with its paralog, *TUBB2A*, sharing a 98% sequence similarity across the coding sequence (https://www.ensembl.org/Homo_sapiens/Gene/Summary?db=core;g=ENSG00000137285;r=6:3223324-3231730, accessed on 18 February 2025). In particular, exon 4, the last coding exon encoding 349 residues in *TUBB2B*, is characterized by >99% homology between the two genes, resulting in low-quality variant calling and preventing reliable mutation detection. Short-read WGS analysis allowed the identification of the c.605T>C *TUBB2B* change, providing a significant advantage on ES due to its capacity to achieve a more uniform sequencing depth and improving alignment accuracy in complex genomic regions. Notably, *TUBB2B* exon 4 is included into the “ENCODE Blacklist” of regions where genome assembly may result in erroneous signals [64]. As proof, the retrospective revision of the reported variants in *TUBB2B* exon 4 highlighted that most of them have been detected by Sanger sequencing (for a complete revision, see [14]). Our findings suggest that ES analysis of these regions might underestimate the real occurrence of causative variants and support the use of targeted approaches in the presence of a strong clinical suspicion.

In conclusion, our findings expand the mutational spectrum of the *TUBB2B*-related CDCBM7 combined with CFEOM, involving a different functional domain of the protein, providing clinical evidence that the p.Ile202Thr *TUBB2B* pathogenic variant can cause CDCBM7 with or without CFEOM as an associated feature.

## Data Availability

The sequencing data that support the findings of this work are available on request from the corresponding authors. The data are not publicly available due to privacy/ethical restrictions.

## Supplementary Materials

The following supporting information can be downloaded at: https://www.mdpi.com/article/10.3390/genes16101182/s1, Figure S1: DNA methylation profiling analysis; Figure S2: Representative chromatograms of the *KDM6A* cDNA sequencing analysis.
