# Supplementary material for: The p.Ile202Thr Substitution in TUBB2B Can Be Associated with Syndromic Presentation of Congenital Fibrosis of the Extraocular Muscles
Source: Genes (Basel). 2025 Oct 11;16(10):1182. doi: 10.3390/genes16101182 (PMC12563659; doi:10.3390/genes16101182)
Supplement: Supplementary file 1 [file genes-16-01182-s001.zip › genes-3861023-supplementary.pdf]

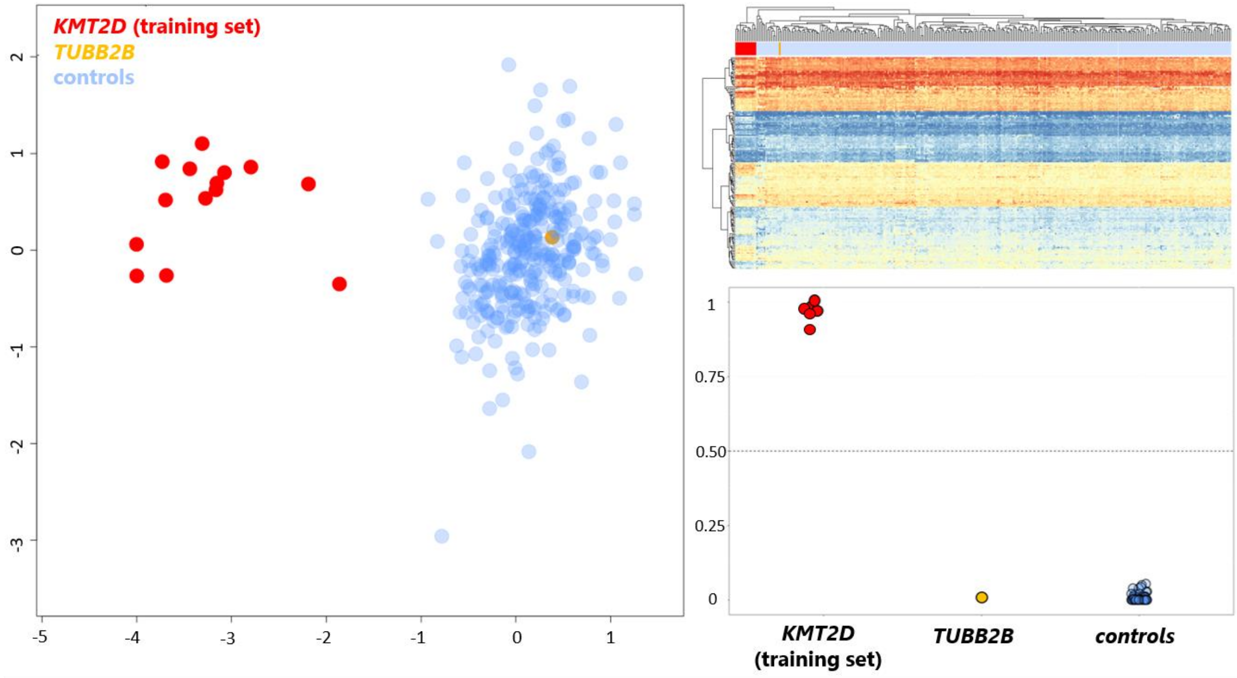

Supplemental Figure S1. DNA methylation profiling analysis.

By using the Kabuki syndrome (KS)-specific DNAm signature, multidimensional scaling plot (left) and hierarchical clustering (right, top) analyses rule out a diagnosis of KS for the proband (orange), which clusters with controls (light blue) and diverges from three individuals with molecularly confirmed KS (red). Plot for SVM probability scores from the developed ML-based classifier, was trained with 14 PB-derived DNA samples from patients with molecularly confirmed clinical diagnosis of Kabuki syndrome and 440 in-house controls including healthy individuals and patients with various neurodevelopmental disorders (light blue). The ML-based classifier refuses a diagnosis of Kabuki syndrome in the proband (orange dot), with high confidence. SVM-score ranges from 0 (= controls) to 1 (= training set).

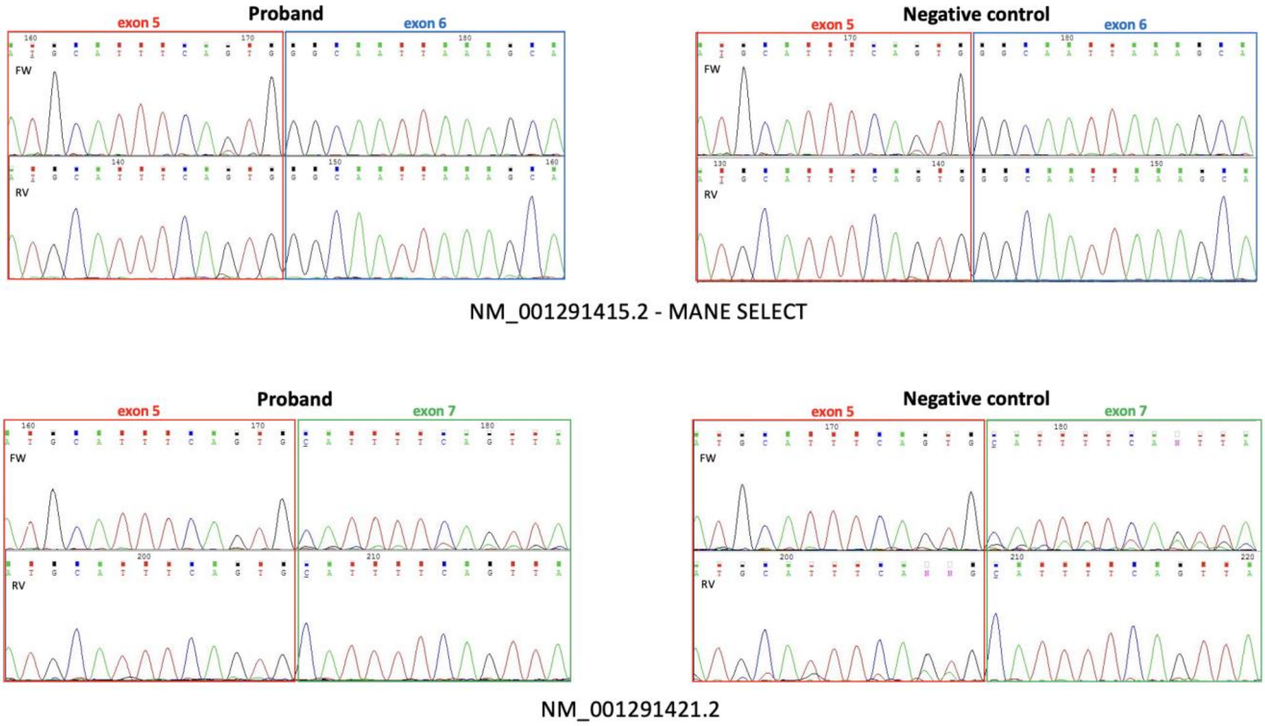

Supplemental Figure S2. Representative chromatograms of the *KDM6A* cDNA sequencing analysis.

Upper panel: Both the proband and the negative control display the canonical exon 5–exon 6 junction, with no evidence of aberrant splicing of the MANE Select transcript (NM_001291415.2).

Lower panel: An alternative transcript (NM_001291421.2) lacking exon 6 is detected in both samples, with the expected exon 5–exon 7 junction clearly demonstrated, indicating that exon 6 skipping represents a physiological isoform rather than a pathogenic splicing event.
